# The Function of SUMOylation and Its Role in the Development of Cancer Cells under Stress Conditions: A Systematic Review

**DOI:** 10.1155/2020/8835714

**Published:** 2020-11-13

**Authors:** Qi Zhao, Ying Ma, Zugui Li, Kexin Zhang, Minying Zheng, Shiwu Zhang

**Affiliations:** ^1^Department of Pathology, Tianjin Union Medical Center, Tianjin, China; ^2^Tianjin Medical University, Tianjin, China; ^3^Department of Spine Center, Tianjin Union Medical Center, Tianjin, China; ^4^Graduate School, Tianjin University of Traditional Chinese Medicine, Tianjin, China; ^5^Nankai University School of Medicine, Nankai University, Tianjin, China

## Abstract

Malignant tumors still pose serious threats to human health due to their high morbidity and mortality. Recurrence and metastasis are the most important factors affecting patient prognosis. Chemotherapeutic drugs and radiation used to treat these tumors mainly interfere with tumor metabolism, destroy DNA integrity, and inhibit protein synthesis. The upregulation of small ubiquitin-like modifier (SUMO) is a prevalent posttranslational modification (PTM) in various cancers and plays a critical role in tumor development. The dysregulation of SUMOylation can protect cancer cells from stresses exerted by external or internal stimuli. SUMOylation is a dynamic process finely regulated by SUMOylation enzymes and proteases to maintain a balance between SUMOylation and deSUMOylation. An increasing number of studies have reported that SUMOylation imbalance may contribute to cancer development, including metastasis, angiogenesis, invasion, and proliferation. High level of SUMOylation is required for cancer cells to survive internal or external stresses. Downregulation of SUMOylation may inhibit the development of cancer, making it an important potential clinical therapeutic target. Some studies have already begun to treat tumors by inhibiting the expression of SUMOylation family members, including SUMO E1 or E2. The tumor cells become more aggressive under internal and external stresses. The prevention of tumor development, metastasis, recurrence, and radiochemotherapy resistance by attenuating SUMOylation requires further exploration. This review focused on SUMOylation in tumor cells to discuss its effects on tumor suppressor proteins and oncoproteins as well as classical tumor pathways to identify new insights for cancer clinical therapy.

## 1. Introduction

The cells in our body are exposed to various stimuli from external or internal environments. The appropriate responses of cells to these stimuli are key for proliferation, apoptosis, and differentiation. When these responses are dysregulated, cellular development is no longer controlled, which may lead to tumor development [1, 2]. Compared to normal cells, tumor cells have undergone increased levels of stress arising from hypoxia, genotoxicity, poor nutrients, and inefficient waste removal [3]. The posttranslational modification (PTM) by small ubiquitin-like modifier (SUMO), namely, SUMOylation, has been a hotspot in tumor studies. Numerous stresses can exert a profound effect on cellular SUMOylation [3, 4]. SUMOylation mainly occurs in the lysine residues of proteins [5], which may act as competitors or prerequisites for ubiquitination and play an important role in maintaining protein stability and improving the stress capacity of cells [6–9]. The balance between SUMOylation and deSUMOylation is a dynamic process [3] to directly regulate cellular responses to different kinds of biotic or abiotic stresses. Changes in intracellular and extracellular environments such as the surrounding temperature, osmotic pressure, oxygen concentration, and oxidative status can result in rapidly accelerated SUMOylation, which may protect cells from damage by various stimuli [10, 11]. To illustrate the importance of SUMOylation in carcinoma, we discuss its roles in several cancers and explore the potential mechanisms by which SUMOylation influences cancer. SUMOylation is a key point in the dysfunction of tumor suppressor proteins and oncoproteins as well as some cancer-related pathways that are common in tumors.

## 2. Function of the SUMO Family Members

The various PTM of proteins includes phosphorylation, acetylation, glycosylation, ubiquitination, and SUMOylation [12]. SUMOylation was first reported in the 1990s [13, 14]; since that time, its functions have been studied in various diseases by regulating the expression and function of different proteins [15]. Four mammalian members of the SUMO family, SUMO-1, SUMO-2, SUMO-3, and SUMO-4 [8, 16, 17], are highly conserved in all eukaryotes [18]. SUMO-1 is a protein with 101 amino acids with a molecular weight of 11.6 kDa. SUMO-2 and SUMO-3 are homologous except for three N-terminal residues but share only about 48% sequence identity with SUMO-1 [19]. SUMO-1, SUMO-2, and SUMO-3 have similar three-dimensional structures. SUMO-1 mainly participates in normal cellular physiology, whereas SUMO-2 and SUMO-3 are mainly associated with the cell stress response. SUMO-4, another SUMO paralogue, shares 86% sequence homology with SUMO-2 and SUMO-3; however, its function remains enigmatic as it may be nonconjugated under normal physiological conditions [6, 20].

Similar to ubiquitin and many other ubiquitin-like proteins (Ubl proteins), all SUMO family members firstly need a protease to transform the immature precursors into the mature form. The C-terminal diglycine in mature SUMO family members is necessary for efficient adenylation by SUMO E1 (SUMO activating enzyme). SUMO E1 is an ATP-dependent enzyme contains SUMO activating enzyme subunits 1 and 2 (SAE1 and SAE2). SAE1 and SAE2 can form a heterodimer [21]. Activated family members combined with SUMO E1 to form an E1 ~ SUMO thioester on a conserved Cys of E1 enzyme; then, activated SUMO family members were transferred from the conserved Cys on E1 to SUMO E2 (SUMO conjugating enzyme) to form an E2 ~ SUMO thioester. SUMO E2 can interact with some substrates directly to transfer SUMO to the Lys residue of substrate, and SUMO E3 (SUMO ligase) can make this direct interaction more efficient. The E2 ~ SUMO thioester and the substrate can be recruited by SUMO E3. When the SUMO E2 interacts with substrate directly, SUMO E3 can also stimulate SUMO E2 to release SUMO and enhance the conjugation between SUMO and substrate [20].

An ubiquitin-conjugating enzyme 9 (Ubc9) is the only SUMO E2 in mammals. It is also directly involved in selecting and can bind directly to the specific SUMO targets that characterize SUMOylation consensus sites (*ψ*-K-X-E, *ψ*: a hydrophobic amino acid, K: the acceptor lysine, X: any amino acid, E: Glu). The two prerequisites for a protein to be SUMOylated are the direct interaction with the Ubc9-SUMO thioester and the recognition of a specific SUMO ligase in proximity with Ubc9. In either case, an acceptor lysine residue with access to Ubc9-SUMO is key and can be achieved via three mechanisms: (a) an acceptor lysine located in a short motif directly recognized by Ubc9 (SUMOylation consensus site); (b) the target protein contains a SUMO interaction motif (SIM) which can recruit the Ubc9-SUMO thioester, and the lysine near the SIM can be modified. SIM-dependent SUMOylation can modify multiple sites including lysine residues that are not contained in the consensus motifs; and (c) since lysine residues in some target proteins cannot be reached by the Ubc9-SUMO thioester, E3 ligase acts like a bridge to interact with the target protein and the Ubc9-SUMO thioester. In this last condition, known as E3 ligase-dependent SUMOylation, the acceptor lysine is determined by the E3 ligase. These mechanisms can indicate the target selection and specificity of SUMOylation [20].

**Figure 1:**
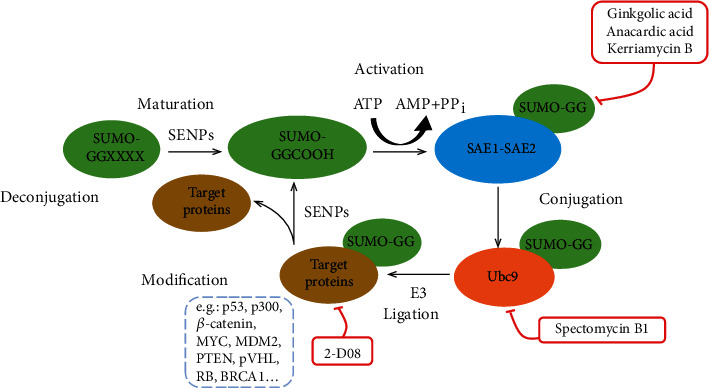
The SUMOylation pathway. All SUMO paralogues are synthesized as preproteins which are first cleaved by *sen*trin-specific proteases (SENPs) and then exposed to the carboxy-terminal diglycine (GG) motif. By consuming an ATP for activation by E1 (SAE1-SAE2), resulting in formation of a SUMO E1 thioester complex. The formation of a SUMO E1 thioester complex can be blocked by ginkgolic acid, anacardic acid, and kerriamycin B. SUMO is transferred to SUMO E2 and linked to a thioester, which can be inhibited by spectomycin B1. SUMO can be directly transferred to the target protein by Ubc9, or sometimes SUMO E3 ligases are also required to connect SUMO to the target proteins at their lysine residues, which can be inhibited by 2-D08 and reversed by SENPs.

SUMOylation can be reversed (deSUMOylated) by SUMO proteases. The balance between SUMO conjugation and deconjugation exerts a profound influence on physiological function in the cell [22, 23]. The two functions of SUMO proteases include the maturation of SUMO family members and the removal of SUMO from target proteins. The deSUMOylation rates determine the steady state of target protein SUMOylation. Many related *sen*trin-specific proteases (SENPs) in vertebrates have been identified on the basis of sequence similarity [24–26]. Cysteine proteases include the CA, CD, CE, and other clans; among these, SENPs belong to the CE clan. The SUMO-specific SENP family includes six proteins: SENP-1, SENP-2, SENP-3, SENP-5, SENP-6, and SENP-7, which have conserved C-terminal catalytic domains and unique substantial N-terminal regions that differ from those of other proteins in genomic databases [23]. Instead of degrading the targets, SENPs release the precursors from C-terminal extensions to liberate SUMO from targets. SUMO can be recycled for the next SUMOylation [27]. SENP-1 and SENP-2 are involved in maturation and deconjugation of SUMO-1 and SUMO-2/3. SENP-3 and SENP-5 are much more specific for SUMO-2/3 than SUMO-1 and can remove SUMO-2/3 from substrate proteins [24]. SENP-5 is important for SUMO maturation; however, the function of SENP-3 in pro-SUMO processing remains unclear due to the difficulty in maintaining purified deSUMOylation enzymes [28]. SENP-1, SENP-2, SENP-3, and SENP-5 mainly deconjugate monoSUMO-1 or monoSUMO-2/3, whereas SENP-6 and SENP-7 favor polySUMO-2/3 over monoSUMO-2/3 [29–31]. The recently discovered deSUMOylation isopeptidase 1 (DeSI1) appears to have high target specificity for Zinc finger and BTB domain-containing protein (BTB-ZF); however, the properties of its paralogue deSUMOylation isopeptidases 2 (DeSI2) remain undefined [32]. Ubiquitin-specific protease-like 1 (USPL1) is another newly discovered SUMO protease that preferentially deconjugates SUMO-2/3 [33] (Figure 1).  Table 1 lists the members of the SUMOylation pathway.

SUMO is an Ubl protein structurally related to ubiquitin, and the amino acid sequence of SUMO-1 is only 18% identical to ubiquitin. The three-dimensional structure of SUMO-1 is similar to that of ubiquitin. Furthermore, two C-terminal Gly residues could form an isopeptide, which were conserved between ubiquitin and SUMO-1 [34]. However, SUMO has a unique N-terminal extension which is not present in ubiquitin. In addition, the distribution of surface charged residues in SUMO-1, -2, -3, and -4 differed from that in ubiquitin and other Ubl proteins [35]. These differences may associate with the function of SUMOylation. Unlike the protein degradation induced by ubiquitin modification, SUMOylation is generally related with the stability of protein. Furthermore, SUMOylation mainly targets nuclear proteins and affects the transcription regulation, DNA repair, and chromatin structure. SUMOylation can compete with ubiquitination or other PTMs occurred at Lys residues. SUMO-modified nuclear factor kappa light chain enhancer of activated B cells inhibitor *α* (I*κ*B*α*) showed increased stability by competing with ubiquitin-induced proteasome degradation [35]. SUMO-modified proliferating cell nuclear antigen (PCNA) participated in mediating DNA repair during cell replication. Ubiquitination at the same lysine residue can also promote DNA repair. Monoubiquitination of PCNA facilitated translational DNA repair and polyubiquitination accelerated error-free DNA repair [36].

Phosphorylation is another kind of PTM. Ubc9 phosphorylated at serine 71 could promote its stability and the level of SUMOylation in the liver, colon, and breast cancer cells [37]. Some proteins can be SUMOylated in the phosphorylation-dependent manner. These substrates are mainly transcriptional regulators and usually have a phosphorylation-dependent SUMOylation motif, which is composed of a SUMO consensus site and an adjacent proline-directed phosphorylation site. The transactivation ability of heat-shock factor 1 (HSF1) and HSF4b could be inhibited through the phosphorylation-dependent SUMOylation [38]. SUMOylation can also promote the phosphorylation of proteins. Protein tyrosine kinase 2 (PYK2) could be autophosphorylated at tyrosine 402, which enhanced the interaction with proto-oncogene tyrosine-protein kinase Src and the activation of Src-PYK2 complex. SUMOylation of PYK2 mediated by the protein inhibitors of activated STAT 1 (PIAS1) or PIAS4 could enhance the autophosphorylation without the upstream stimulus. SUMO-modified PYK2 promoted the migration of breast cancer cells through the Src, paxillin, and ERK1/2 pathway, which suggested that SUMOylation played a critical role in the tumor development [39]. Acetylation at lysine is another PTM and can also affect SUMOylation. Acetylation could promote the SUMOylation of histone H4 [40], and SUMOylation could inhibit acetylation by facilitating the deacetylase activity of some deacetylases such as histone deacetylase (HDAC) 1 [41].

SUMOylation affects normal cells in various ways. SUMOylation usually plays a negative role in regulating transcription factor activity by changing the interaction with DNA and chromatin to repress gene expression. Ubiquitin modification involved in transcription factors often leads to gene activation [42]. SUMOylation modifies transcriptional activators, coactivators, repressors, and corepressors. Decreased SUMO attachment to transcription factors such as Elk-1, C/EBPs, c-myb, and STAT-1 could increase transcription activity [43–45]. One possible mechanism of transcriptional repression is SUMO recruitment of other transcription repress factors such as the repressor protein Daxx, PIAS proteins, and HDACs by binding with a promoter. The mechanism of p300 SUMOylation suggests that HDAC6 inhibition can sharply weaken SUMO-dependent transcriptional repression [46]. Secondly, SUMOylation also plays an important role in DNA repair. Thymine and uracil can be removed by thymine-DNA glycosylase (TDG) from mismatched G-T and G-U base pairs. The affinity of TDG binding to the DNA substrate is weakened by SUMOylation. When the DNA repair is incorrect, non-SUMOylated TDG binds to the DNA substrate and excises the incorrect bases. SUMOylation reduces the interaction between TDG and DNA substrates to release TDG into circulation [47]. Thirdly, SUMOylation also plays a critical role in nuclear and subnuclear localization of proteins. For instance, Ran GTPase-activating protein 1 (RanGAP1), which activates the small GTPase Ran, was the first substrate discovered to be modified by SUMO. RanGAP1 is involved in nucleocytoplasmic transport. Unmodified RanGAP1 is located in the cytoplasm. After modification by SUMO, RanGAP1 interacts with RanBP2, one of the nuclear pore complex (NPC), and SUMO E3 ligases [48]. SUMO-RanGAP1 binds tightly to NPC, which is critical for nuclear import. In mammalian cells, RanBP2 has a broad influence on nuclear import induced by SUMOylation [13].

## 3. SUMOylation Is Involved in Different Stress Conditions

Cancer initiation and development involve numerous stresses from hypoxia, nutrient loss, low waste removal efficiency, DNA damage (genotoxic stress), and host immune system response [49]. It has been well documented that many biotic and abiotic stimuli exert profound effects on the cellular SUMOylation. For example, in heat shock-induced SUMOylation, all the SENPs (except SENP6) become inactivated because they are heat-sensitive [50]. Furthermore, the level of unSUMOylated SAE2 is also increased by heat shock. UnSUMOylated SAE2 can transfer SUMO to Ubc9 more efficiently and promote the SUMOylation of target proteins [51]. Upregulated SUMOylation may sensitize cells to various stresses. Several pathways may be involved in the responses of tumor cells to various stresses.

### 3.1. SUMOylation Regulates the Hypoxic Stress of Cancer Cells

**Figure 2:**
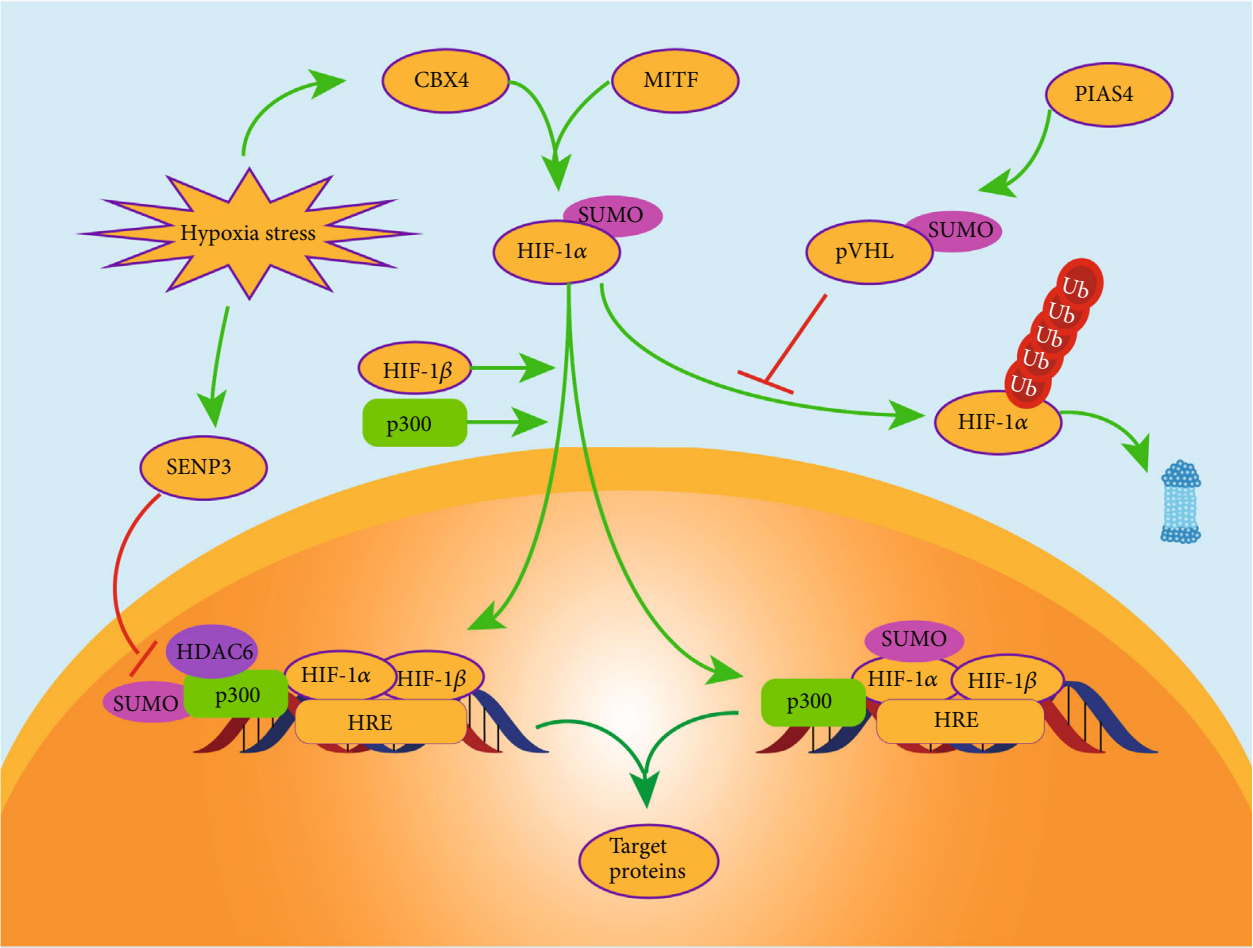
SUMOylation in the hypoxia pathway. SUMOylation plays a positive role in maintaining the stabilization of hypoxia-inducible factor 1-alpha (HIF-1*α*). SUMO-modified von Hippel–Lindau tumor suppressor (pVHL) cannot modify HIF-1*α* by ubiquitination and degrade it through proteasomes. Microphthalmia-associated transcription factor (MITF) and chromobox 4 can upregulate HIF-1*α* and facilitate its nuclear translocation. p300 can be modified by SUMOylation under mildly hypoxic conditions. SUMO-modified p300 can recruit HDAC6 and inhibits the transcriptional activity. This process can be inhibited by SENP3 through deSUMOylating p300 and thus increases the HIF-1-dependent vascular endothelial growth factor (VEGF) expression.

Cancer cells are sensitive to peripheral oxygen concentration and more easily escape cancer therapy and develop resistance in hypoxic conditions [52]. Conventional chemotherapy and radiation therapy are more effective in proliferating cells under well-oxygenated conditions. In hypoxic conditions, the levels of SUMOylation proteins sharply increase in cancer cells, including SUMO-1 [53], the SUMO E3 ligase PIAS4 [54, 55], the SUMO enhancer RSUME [56], and SENP1 [57, 58]. The hypoxia-related signaling pathway is critical for cancer development because it can regulate the transcription of more than 1,500 target genes involved in angiogenesis, epithelial-mesenchymal transition (EMT), cancer stem cells maintenance, metastasis, extracellular matrix remodeling, and immune evasion. Hypoxia-inducible factor (HIF) 1*α* acts as a transcriptional regulator of the adaptive response to hypoxia and is upregulated through SUMO-sensitive microphthalmia-associated transcription factor (MITF) [59]. HIF-1*α* regulates the expression of over 40 proteins including vascular endothelial growth factor (VEGF), glycolytic enzymes, erythropoietin, and so on. These proteins increase oxygen transportation or promote metabolic adaption to hypoxia [60]. The ubiquitin E3 ligase activity of von Hippel-Lindau protein (pVHL) decreases due to SUMOylation by PIAS4 and the stabilization of HIF-1*α* increased [61]. It has been reported that chromobox 4, a SUMO E3 ligase, can enhance the SUMOylation of HIF-1*α* at K391 and K477 through SIM-dependent way in hepatocellular carcinoma (HCC) and improve the expression of VEGF and angiogenesis [62]. However, the SUMOylation of HIF-1*α* does not always positively affect the hypoxia pathway. During SENP1 deficiency in mouse placental development, SUMOylated HIF-1*α* suffers prolyl hydroxylation-independent degradation, which indicates an attenuated HIF-1*α* response to hypoxia even with increased HIF-1*α* stabilization [63] (Figure 2). Moreover, in prostate cancer, a mild increase in reactive oxygen species (ROS) mediates SENP3 stability and transportation from the nucleoli to the nucleoplasm, a redistribution that contributes to the increased transcription of HIF-1*α* to stimulate tumor angiogenesis. Intriguingly, p300 rather than HIF-1*α* participates in this process; in other words, SENP3 enhances the HIF-1 expression by removing SUMO-2/3 from p300 under oxidative stress [64]. Additionally, activating enhancer binding protein 2 alpha (TFAP2A) plays a critical role in tumorigenesis, tumor invasion, and metastasis of many cancers including breast cancer, melanoma, and glioma. It is reported that TFAP2A could be modified by SUMO-2/3 and interacted with HIF-1*α* and HIF-2*α* [65]. However, the inhibition of TFAP2A SUMOylation under hypoxic condition can improve the transcriptional activity of HIF-1*α* and promote cancer cell survival [65]. Taken together, SUMOylation affects multiple steps of hypoxia pathway, and the effect of SUMOylation on hypoxia pathway should be considered on context-specific conditions.

### 3.2. Genotoxic Stress and SUMOylation

**Figure 3:**
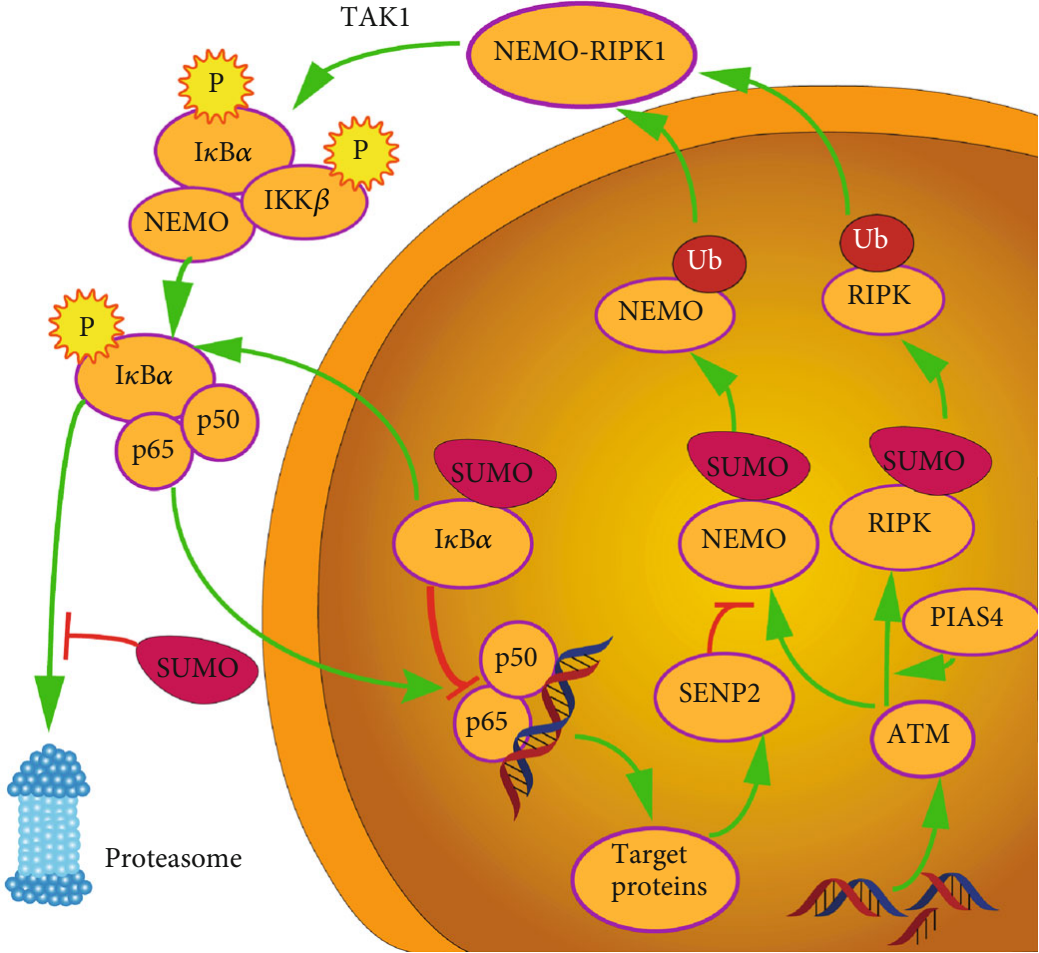
SUMOylation in the nuclear factor kappa-light-chain-enhancer of activated B cells (NF-*κ*B) pathway. The NF-*κ*B pathway is activated by DNA damage. Unlike the canonical NF-*κ*B pathway, NF-*κ*B signaling activated by genotoxicity requires ataxia-telangiectasia mutated (ATM) kinase, which results in the SUMOylation of NF-*κ*B essential modulator (NEMO). Protein inhibitor of activated STAT 4 promotes the SUMOylation of NEMO and RIPK. After SUMOylation, NEMO and receptor-interacting serine/threonine-protein kinase 1 (RIPK) are subsequently ubiquitylated and can then translocate into the cytoplasm to form a complex to recruit transforming growth factor-beta-activated kinase 1 (TAK1). The TAK1 kinase phosphorylates the inhibitor of nuclear factor-*κ*B (I*κ*B) kinase alpha (IKK*α*) and IKK*β*. Following I*κ*B*α* phosphorylation, p65 and p50 are released and redistributed to nuclear and transcript target proteins. SUMOylation can decrease the NF-*κ*B-dependent gene expression by stabilizing I*κ*B*α* through competing with ubiquitylation and promoting the nuclear location of I*κ*B*α*. SENP2 induced by the activated NF-*κ*B pathway can deSUMOylate NEMO and inhibit the subsequent ubiquitylation.

Antiapoptotic mechanisms are important for cancer cells under genotoxic stress induced by chemotherapy. Nuclear factor kappa light chain enhancer of activated B cell (NF-*κ*B) signaling is a crucial pathway involved in antiapoptotic mechanisms, which are critical hallmarks for tumor development and which mediate cell susceptibility to apoptotic signaling [66–68]. Genotoxicity is a strong inducer of NF-*κ*B activation. Ataxia telangiectasia mutated kinase (ATM), a type of serine/threonine-protein kinase, acts as a DNA damage sensor that activates checkpoint signaling when double-strand breaks, apoptosis, and genotoxic stresses occur. PTM of NF-*κ*B essential modulator (NEMO) is indispensable to link the cellular genotoxic response to NF-*κ*B via the ATM kinase [69, 70]. NEMO contains a C-terminal zinc finger domain necessary for PTM in the nucleus when exposed to DNA damage such as SUMOylation [68]. SUMOylation of NEMO occurs at lysine residues 277 [68] by SUMO-1 and 309 by the SUMO E3 ligase (PIAS4) [71, 72]. Mutation of K277 can decrease NF-*κ*B activation induced by DNA damage [71, 73]. SUMOylation contributes to NF-*κ*B nuclear localization in response to DNA damage, which is consistent with other SOMO-1 substrates [61, 74] (Figure 3). In contrast, increased NF-*κ*B activation leads to increased SENP2 and SENP6 expression, which weakens the inhibit effect of nuclear factor-*κ*B (I*κ*B) kinase (IKK) via the deSUMOylation of NEMO [75, 76]. Furthermore, the noncanonical I*κ*B kinase IKK*ε* (IKKi) is another SUMO-modified protein involved in NF-*κ*B pathway. Upon genotoxic stress, IKKi is SUMOylated by TOPORS at K231, which functions as a SUMO E3 ligase. SUMO-modified IKKi is critical for the phosphorylation and activation of nuclear substrates like NF-*κ*B p65 and contributes to the antiapoptotic function of NF-*κ*B in response to genotoxic stress [73]. The NF-*κ*B pathway is also involved in proinflammation [77, 78]. After most cancer cells are killed, chemotherapy or radiation therapy creates aseptic inflammation due to the products of tumor cell disintegration remaining in the blood, which stimulates the NF-*κ*B pathway to help surviving cancer cells to endure the toxicity of chemoradiotherapy. DNA damage caused by chemotherapy leads to activation of ATM-NEMO-IKK signaling. SUMOylation plays a positive role in the NF-*κ*B pathway.

### 3.3. Senescence and SUMOylation

Senescence, the permanent cell cycle arrest, is a common cellular response upon stresses and is also recognized as a critical tumor suppressive mechanism [79]. Senescent cells affect neighboring nonsenescent cells by senescence-associated secretory phenotype (SASP), which involves the production of extracellular enzymes and proinflammatory cytokines. SASP not only contributes to tumor development and metastasis but also improves antitumor immunity [80, 81].

Chromatin immunoprecipitation and next-generation sequencing (ChIP-seq) was used by Neyret-Kahn et al. to detect the SUMOylation status of chromatin-associated proteins in senescent fibroblasts induced by oncogenic stress and proliferating fibroblast. SUMOylation occurs mainly at the promoters of histone protein biogenesis genes, Pol I rRNAs, and Pol III tRNAs and negatively regulates the expression of these genes. The research revealed that genome-wide loss of chromatin associated SUMO reactivity like Ubc9 and PIAS4 E3 ligase compared with nonsenescent fibroblasts. For histone and tRNA genes, SUMOylated chromatin-associated proteins are specifically retained in senescent cells, which indicated that SUMOylation played an important role in restraining the activity of these genes involved in cell proliferation [82]. In addition, the depletion of Ubc9 in primary human fibroblasts can cause a senescence-like growth arrest [82]. Repression of SENP1 can mediated premature senescence of normal human fibroblasts by enhancing p53 transcriptional activity [83]. In conclusion, cellular senescence can be induced by dysregulated SUMOylation, which may exert a profound effect on tumor cells and tumor microenvironment. In the meantime, senescence can affect the SUMOylation of several genes to mediate cell growth.

## 4. SUMOylation of Tumor-Associated Proteins

The activation of oncogenes and the inactivation of tumor suppressor genes are the key steps of tumor initiation. As an important form of PTM, SUMOylation is associated with the regulation of both tumor suppressors and oncoproteins.

P53, the most frequently mutated tumor suppressor protein, usually functions as a transcription factor to regulate apoptosis, proliferation, and senescence. In both vertebrates or invertebrates, p53 proteins regulate a variety of cellular stress response programs including apoptosis [84]. P53 is modified by monoubiquitylation to directly facilitate its interactions with SUMO family members [85]. The mechanism of the ability of SUMOylation to attenuate p53 transcriptional activity may also be associated with acetylation. The interaction between SUMO-1-conjugated p53 and p300 histone acetyltransferase is as efficient as that for the unmodified protein. However, p53-dependent chromatin transcription cannot be activated due to its inability to bind to DNA. P53 is modified by SUMOylation at K386, which inhibits subsequent acetylation by p300. Acetylated p53 alleviates inhibited DNA binding by SUMOylation. When K386 is mutated to K386R, p53 cannot be SUMOylated, and the transcriptional activity is restored. SUMOylation of P53 reduces transcriptional activity by hindering subsequent acetylation and DNA binding [86]. MDM2, as an E3 ligase ubiquitinating P53, is one of the most significant regulators of P53 that promotes the interaction between P53 and the PIASy SUMO E3 ligase. SUMOylation strengthens the P53-MDM2 interaction and degrade P53 [86]. Furthermore, the proto-oncogene Ski is overexpressed in numerous cancers such as colorectal, leukemia, pancreatic, and gastric cancers. Ski negatively regulates P53 by enhancing MDM2 SUMOylation to stabilize MDM2 [87].

Other tumor suppressors include pVHL and breast cancer 1 protein (BRCA1), both of which are E3 ubiquitin ligases. In renal cell carcinoma, pVHL oligomerization by PIAS4-mediated SUMOylation enhances HIF-1*α* stabilization to promote cancer cell migration, clonogenicity, and migration [54]. Mutation of BRCA1 is positively related with breast and ovarian cancer. It functions as a ubiquitin ligase in participating in DNA damage response, and the SUMOylation of BRCA1 can increase its ligase activity [88, 89].

Phosphatase and tensin homolog (PTEN) is a tumor suppressor that is usually mutated in human cancers [90, 91] and regulates the phosphatidylinositol-3-kinase (PI3K)/AKT pathway via the dephosphorylation of phosphatidylinositol-3,4,5-triphosphate (PIP3) [92]. PTEN negatively regulates the PI3K signaling pathway in the cytoplasm and accumulates in the nucleus to control DNA repair and sensitivity of cancer cells to genotoxic stress. SUMOylation is necessary for the function of PTEN in DNA damage repair. PTEN can be SUMOylated at K254 site. Mutation-type PTEN with K254R (cells were mutated from lysine to arginine to inhibit the PTEN SUMOylation) was exposed to irradiation to create a genotoxic stress. Bassi et al. found that TP53-binding protein 1, a kind of protein which is related with the double-strand breaks of DNA, had largely resolved in wild-type PTEN cells but not in K254R cells after 24 h irradiation treatment. DNA repair protein RAD51 cannot be recruited to the DNA damage sites in K254R cells, which indicates the failure of homologous recombination- (HR-) based repair. The level of SUMO-PTEN began to decrease after treated by irradiation for 1 h and recovered 8 h later, which is consistent with the appearance of DNA damage induced by irradiation. This process can be blocked through the inhibition of ATM. Cancer cells with nuclear location of SUMO-PTEN are more resistant to DNA damage than cells without nuclear PTEN [93]. Additionally, SUMO-1 modification of PTEN at K266 affects PTEN membrane association to downregulate the phosphatidylinositol-3 kinase/AKT pathway by facilitating the combination of PTEN and the electronegative phosphorylation membrane to dephosphorylate PIP3, which suppresses anchorage-independent tumorigenesis and cell growth *in vivo* [94].

The retinoblastoma tumor suppressor protein (pRB) traditionally functions as a key regulator of the cell cycle, cell proliferation, and cell differentiation [95–97], similar to P53 [98]. pRB is often inactivated in tumors, and its inactivation results in uncontrolled cancer cell proliferation and loss of anticarcinoma mechanisms. In other words, E2F transcription factor overexpression promotes cell cycle transition from the G1 to S phase. Underphosphorylated pRB, the activated form of pRB, interacts with the E2F transcriptional factors and represses the transcriptional activity, resulting in cell cycle arrest at G1, whereas phosphorylated pRB disrupts the interaction between pRB and E2F. Cell cycle-related proteins regulate the function of pRB, which commonly mutated in various types of cancers. pRB dysregulation is an important event in tumor initiation in various types of cancers. The inactivation of pRB and P53 genes is increasingly frequent in advanced tumors and may explain why advanced tumors are more resistant to recurrent clinical therapy [98]. The main combining region of pRB is the pocket domain, and the pocket domain is the central of pRB [99–101]. Various cellular pRB-binding partners and oncoproteins can bind to this region; the characteristics of these partners and proteins are referred to as the LxCxE sequence [102]. SUMO family members preferentially interact with hypophosphorylated pRB, the activated form of pRB. pRB is modified by SUMOylation at a distinct residue (K720) within the pocket region of B-box. SUMOylation of pRB disrupts the combination of pRB with the low-affinity pRB-binding partners and slightly inhibits the transcriptional repression mediated by pRB. For example, the repression of E2F mediated by SUMO-deficient mutation pRB^K720R^ is moderately lower than that of the wide-type pRB, which shows that loss of SUMOylation can improve the repressive capacity of pRB [103]. In conclusion, SUMOylation plays different roles in different tumor suppressor proteins and is required for cancer cells to survive in stressful environments.

S100A4 is a member of the small Ca^2+^-binding S100 superfamily and is associated with tumorigenesis, metastasis, proliferation, invasion, and angiogenesis [104]. S100A4 in the microenvironment secreted by cancer cells interacts with stromal cells around tumor cells to promote tumor metastasis [105, 106]. In addition to its important role in the cytoplasm, S100A4 can translocate to the nucleus to act as a transcriptional factor. This nuclear translocation was first discovered in colorectal cancer in 2003; the researchers reported that the nuclear translocation was positively associated with advanced stages of colorectal carcinoma [107] and cancer invasion in epithelial ovarian carcinoma by increasing RhoA levels [108]. The nuclear translocation of S100A4 is associated with SUMOylation modification. Treatment of choriocarcinoma (CCA) cells with ginkgolic acid, a SUMOylation inhibitor, results in low levels of the nuclear S100A4 expression that inhibit CCA cell invasion and proliferation [109]. In addition, the nuclear expression of S100A4, as a nuclear transcription factor, regulates the expression of matrix metalloproteinase- (MMP-) 13 [110] and degrades the extracellular matrix to promote cancer invasion. Abnormal nuclear location of S100A4 can be viewed as a signal for tumor invasion, and downregulation of SUMOylation may help in choriocarcinoma therapy.

*β*-catenin is the key protein of the WNT signaling pathway [111]. High levels of *β*-catenin SUMOylation are associated with poor prognosis [112]. In multiple myeloma (MM), ubiquitin-proteasomal-mediated degradation of *β*-catenin is reduced, and *β*-catenin stabilization is increased via SUMOylation, resulting in the EMT of tumor cells and poor patient prognosis [113–115]. Cyclin D1 binds with CDK4 or CDK6 to form activated complexes and then phosphorylates RB to drive cell cycle progression from G1 to S1 phase [116]. *β*-catenin, which regulates the canonical Wnt pathway, can form complexes with lymphoid enhancer factor/T cell factor (LEF/TCF) that target motifs within the gene promoter of CCND1 (the gene encoding cyclin D1) to upregulate the cyclin D1 expression [117] SUMOylation stabilizes *β*-catenin and promotes its nuclear localization and positively mediates cyclin D1 expression. Cyclin D1 combines with CDK4 and is translocated to the nucleus during the G1 phase [118]. Glycogen synthase kinase-3*β* (GSK-3*β* kinase) enters the nucleus during the G1/S transition to phosphorylate cylinD1 [119], which is then exported to the cytoplasm. Cyclin D1 in the cytoplasm is ubiquitinated and degraded by the proteasome [120]. In summary, SUMOylation modification of oncoproteins can promote cancer development and is negatively related with the prognosis of patients.

## 5. SUMOylation in Malignant Tumor Development and Chemoresistance

Aberrant expression of SUMOylation family members and substrates is observed in numerous cancer types and helps cancer cells to maintain their capacity for differentiation, proliferation, and responses to stresses exerted by intrinsic or extrinsic stimuli. Besides playing an important role in tumor proliferation and progression, SUMOylation is also critical for tumor resistance to radiotherapy and chemotherapy.

### 5.1. The Role of SUMO E1 Activating Enzyme in Cancers

SUMO E1 activating enzyme is a heterodimer formed by SAE1 and SAE2, and the expression of SUMO E1 can be detected in many kinds of malignant tumors. The overexpression of SAE1 is correlated with the poor prognosis of patients, which suggests that SUMOylation may involve in the progression of malignant tumors. Additionally, the transcriptional level of SAE1 was significantly correlated with lymph node metastasis by comparing the gene expression difference between lymph node-positive and lymph node-negative lung adenocarcinomas [121]. SAE2 is another important subunit of SUMO E1 activating enzyme and is essential for the SUMOylation of numerous proteins. SAE2 was highly expressed in small cell lung cancer (SCLC) with c-Myc overexpression. Inhibition of the SAE2 expression in SCLC could decrease the proliferation of cancer cells and increase the sensitivity of cancer cells to cisplatin and etoposide. SAE2 may also be a potent therapeutic target for SCLC with a high expression of c-Myc [122]. Myc oncogenic transcription factors including c-Myc, N-Myc, and L-Myc could result in a hyper-SUMOylation state, and the levels of SAE1, SAE2, and Ubc9 were upregulated in Myc-induced lymphoma [123]. Inhibition of SUMOylation by genetic means or small molecule inhibitors can inhibit Myc-induced proliferation. Thus, targeting SUMOylation may represent a new therapy for Myc-induced lymphoma [123].

### 5.2. The Role of SUMO E2 Conjugating Enzyme in Cancers

Ubc9 is the only SUMO E2 conjugating enzyme to catalyze the formation of Ubc9-SUMO thioester. The expression of Ubc9 in primary colon and prostate cancer increased compared with their normal tissue counterparts. However, in metastatic breast, prostate, and lung cancer, it is decreased in comparison with their corresponding normal and primary adenocarcinoma tissues [124]. The high level of Ubc9 was also be found in breast cancer (especially in luminal type), and PTM of Ubc9 can be mediated by microRNA. The miR-30 family, such as miR-30e, was reduced in tumor and negatively regulated the expression of Ubc9 [124, 125]. The expression of Ubc9 was associated with the development and chemoresistance of breast cancer and the patients' prognosis [126]. The mechanism of Ubc9 regulating chemoresistance still remains unclear. Previous studies showed that Ubc9-mediated chemoresistance depends on Bcl-2. Downexpression of Ubc9 in MCF-7 cells inhibited the tumor growth in nude mice through increasing Bcl-2-associated apoptosis [127]. In melanoma, Ubc9 was overexpressed in lymph node metastatic foci and played an important role and protected advanced-stage melanomas from chemotherapy-induced apoptosis. Inhibition of Ubc9 can make melanoma cells sensitive for chemotherapeutic drugs [128]. Furthermore, hepatic S-adenosyl methionine (SAMe) is decreased in HCC, but Ubc9 is increased. After the treatment of liver cancer cells with the SAMe or its metabolite 5′-methylthioadenosine (MTA), the protein level of Ubc9 is reduced but not the mRNA level. Cell division cycle 2 (Cdc2) could phosphorylate Ubc9 at serine 71 in liver cancer cells and enhance its SUMOylation ability, which can be inhibited by SAMe. It is reported that hyperphosphorylated Ubc9 represented a mechanism to maintain a high level of SUMOylation in liver cancer, which could be repressed by the SAMe and MTA [37].

### 5.3. The Role of SUMO E3 Ligases in Cancers

Unlike the SUMO E1 and E2 which are required for the SUMOylation of all the substrates, the effects of SUMO E3 in cancer are more restricted and specific than SUMO E1 and E2. The expression of PIAS1 is substantially higher in prostate cancer than in normal tissues. Downregulation of PIAS1 impairs colony formation and proliferation of prostate cancer cells through the p21-dependent cell cycle arrest in the G_0_/G_1_ phase [129]. High level of the PIAS1 expression was also observed in breast cancer, and the knockdown of PIAS1 inhibited tumor growth *in vivo.* A subset of clinic-related genes such as cyclin D2, estrogen receptor, and breast tumor suppressor WNT5A is silenced by PIAS1 through histone modification and DNA methylation which indicates that PIAS1 can regulate tumorigenesis by selectively silencing genes [130]. The tumor suppressor promyelocytic leukemia protein (PML) is modified by SUMOylation by interacting with PIAS1, which promoted its ubiquitin-mediated degradation in acute promyelocytic leukemia and thus attenuated its tumor suppressor functions [131]. PIAS1 is also essential to the SUMOylation of PML in non-small-cell lung cancer (NSCLS) [131]. Hypoxia is a common stimulus for cancer and promotes metastasis by regulating EMT of cancer cells. SIRT1 can negatively regulate ovarian cancer metastasis by inhibiting EMT. However, the expression of SIRT1 is downregulated under hypoxic stress because the SUMO E3 ligase PIAS4 can prevent the occupancy of the transcriptional activator Sp1 on the promoter of SIRT1 gene, and the expression of SIRT1 can be restored through the knockdown of PIAS4. PIAS4 was positively correlated with the malignancy of human ovarian cancer [132]. Furthermore, PIAS4 could promote the activity of hypoxia signaling pathway by interacting with VHL, which leads to VHL SUMOylation and impairing VHL's function in pancreatic cancer cells [133].

### 5.4. The Role of SUMO-Specific Proteases in Cancers

As we described above, SUMOylation is a reversible process, and SENPs promote the recycle of the next SUMOylation. The increased SENP1 mRNA level was observed in the urine of bladder cancer patients and correlated with cancer recurrence [134]. SENP1 is also overexpressed in MM. The members of NF-*κ*B pathway, including P65 and inhibitor protein I*κ*B*α*, played an important role in regulating the survival and proliferation of MM cells. Inhibition of SENP1 can downregulate IL-6-induced P65 and I*κ*B*α* phosphorylation, which make NF-*κ*B pathway inactivated. Overexpressed SENP1 promoted the proliferation of MM cells by positively regulating the NF-*κ*B pathway [135]. In human prostate cancer, the upregulation of SENP1 was significantly correlated with poor biochemical-free survival. The expression level of SENP1 was an independent prognostic factor for biochemical recurrence after radical prostatectomy [136]. Neuroblastoma (NB) is an embryonic solid tumor and accounts for 11% of childhood cancers. Overexpressed SENP1 promoted the invasion and migration of NB cells by regulating the expression of cadherin 1, MMP9, and MMP2 [137]. However, SENP2 functions as a tumor suppressor in bladder cancer by limiting the expression of MMP13 and inhibiting the invasion and migration of bladder cancer [138]. SENP3 was also found overexpressed and correlated with the differentiation of human oral squamous cell carcinoma (OSCC). A modest increased ROS can induce SENP3 translocation from the nucleoli to the nucleoplasm, which may play a critical role in the development of OSCC under oxidative stress [139]. The low expression of SENP5 is correlated with a good prognosis in patients with breast cancer. The type I transforming growth factor-*β* (TGF*β*RI) can be modified by polySUMO and leads to its ubiquitination and degradation. SENP5 stabilizes TGF*β*RI by interrupting its polySUMO and degradation. MMP9 was critical in TGF*β*-induced invasion and depletion of SENP5 resulted in a dramatic reduction of MMP9, which indicates that SENP5 regulated the invasion of breast cancer by TGF*β*-induced MMP9 [140] (Table 2).

Elevated levels of SUMO E1 activating enzymes, SUMO E2 conjugating enzymes, and SUMO E3 ligases are prevalent in cancers. They are usually positively associated with different cancer stages and promote malignancy [126, 150, 151]. The levels of SUMOylation proteases (SENPs) are also enhanced in neuroblastoma and oral squamous cell carcinoma (OSCC). The SENP1 expression is higher in metastatic NB tissues than that in primary NB tissues, which may be due to the regulation of SENP1 in the expression of MMP-2 and MMP-9. SENP3 is upregulated in OSCC, and its stability is regulated by ROS. SENP3 translocates to the nucleoplasm to initiate certain transcription factors, including PML, by deSUMOylation [137, 139]. The balance between SUMOylation and deSUMOylation is essential for cells to maintain physiological functions. Disruption of this balance by upregulation of SUMOylation or deSUMOylation may contribute to cancer development and progression.

## 6. SUMOylation Inhibitors and Therapeutic Prospective

Ginkgolic acid and anacardic acid are both small-molecule inhibitors of SUMOylation. SUMOylation *in vitro* can be inhibited completely by ginkgolic acid at 10 *μ*M. Anacardic acid is a histone acetyltransferase inhibitor with the similar structure to ginkgolic acid and can also inhibit SUMOylation *in vitro.* Mechanistically, the carboxylic acid of ginkgolic acid was essential for the direct and specific binding with SUMO E1, and ginkgolic could interrupt the formation of E1-SUMO thioester complex [152]. Blocking the SUMOylation in breast cancer cells by using ginkgolic acid and depleting SUMO1 and UBC9 can induce autophagy-mediated cell death in a tribbles pseudokinase 3- (TRIB3-) dependent manner. Inhibition of SUMOylation downregulated the invasion ability of breast cancer cells by impairing the activation of the small GTPase RAC1 [153]. Furthermore, anacardic acid could decrease chemoresistance of AMLs by restoring the expression of the proapoptotic gene DDIT3 and reduce the tumor burden of mice xenografts of AMLs resistant model [154]. Kerriamycin B was another small-molecule inhibitor which also blocks the E1-SUMO intermediate [155]. Spectomycin B1 could inhibit SUMOylation by directly binding with E2 (Ubc9) and subsequent E2-SUMO complex formation [156]. In hormone-dependent breast cancer, the proliferation of cancer cells and tumorigenesis could be inhibited either by exposing to spectomycin B or by silencing the Ubc9 gene [156]. 2-D08 is another small-molecule inhibitor that can block SUMOylation without affecting ubiquitylation. The transfer of SUMO-1 from Ubc9 to substrate was blocked by 2-D08 [157]. Camptothecin facilitated the SUMOylation of topoisomerase 1, which exerted a profound influence on camptothecin resistance [158, 159]. After treating ZR-75-1 cells and BT-474 cells with 2-D08, the level of SUMOylated topoisomerase 1 was decreased [157]. SUMOylation is essential for the development and progression of malignant tumors, especially Myc-driven cancer cells. Therefore, the key enzymes associated with SUMOylation pathway may be targets of the potential clinical therapy for malignant tumors (Table 3).

## 7. Future Perspectives and Conclusions

Compared with normal cells under physiological conditions, cancer cells are exposed to many more stresses induced by chemotherapy or radiotherapy, including genotoxicity, hypoxia, and host immune system response. As a PTM, a high level of SUMOylation in cancer cells can improve the cellular responses to stress by regulating the functions of key tumor-related proteins. Hyper-SUMOylation is required for these cancer cells to survive under stress. Furthermore, SUMOylation also play a role in tumorogenesis. In the KRAS mutant colorectal cancer, the growth of cancer cells can be repressed by the depletion of Ubc9 *in vitro* and *in vivo.* Inhibition of Ubc9 may present a potent therapy for KARS mutant colorectal cancer [160]. Secondly, the inhibition of SUMOylation impairs Myc-dependent tumorigenesis in breast cancer [161], which indicates that SUMOylation can represent a potential clinical therapeutic target. Some studies have begun to explore the possibility of cancer treatment by inhibiting the expression of SUMOylation family members [154] including SUMO E1 [152], or E2 [157]. The dysregulation of SUMOylation affects many biological processes such as proliferation, DNA repair, apoptosis, and survival. The numerous SUMO substrates play various and complicated functions in different kinds of tumor cells. Inhibition of specific SUMOylation events rather than of global SUMOylation may be a potential therapy which needs to be studied in the future.

In summary, tumor cells become more aggressive under internal and external stresses. The prevention of tumor development, metastasis, recurrence, and radiochemotherapy resistance by the perturbation of specific key SUMOylation events requires further exploration.

## Figures and Tables

**Table 1:** Members of the SUMO family.

SUMO family members	*Homo sapiens*
SUMO	SUMO-1, -2, -3, -4
SUMO-activating enzyme E1	SAE1, SAE2
SUMO-conjugating enzyme E2	Ubc9
SUMO-ligase E3	Ranbp2; protein inhibitor of activated STAT (PIAS)1, -2, -3, -4; Nse2Mms21; etc.
SUMOylation proteases	SENPs, DeSI1, DeSI2, USPL1, etc.

**Table 2:** SUMOylation in different kinds of cancers.

Components	Expression	Effects on cancers
SUMO1, SUMO2/3	Upregulated	Silencing the expression of SUMO1 and SUMO 2/3 impairs cell growth and DNA synthesis in glioblastoma [141]. SUMO2/3 is critical for proliferation of Myc-driven lymphoma [123].

SAE1	Upregulated	SAE1 is positively related with lymph node metastasis of lung adenocarcinomas [121]. SAE1 is important for proliferation of Myc-driven lymphoma [123]. SAE1 promotes the progression of hepatocellular carcinoma [1].

SAE2	Upregulated	SAE2 can maintain the malignancy and reduce the chemotherapy sensitivity in SCLC [122]. SAE2 is critical for proliferation of Myc-driven lymphoma [123].

Ubc9	Upregulated	Negatively associated with survival rate of multiple myeloma [112]. Critical for proliferation of Myc-driven lymphoma [123]. Involves in human lung tumorigenesis [142]. Inversely correlated with the sensitivity of chemotherapy and prognosis of breast cancer [126]. Serves as an important molecule in melanoma-positive lymph nodes [128].

PIAS1	Upregulated	Negatively associated with survival rate of multiple myeloma [112]. Promotes breast tumorigenesis by selectively silencing the epigenetic genes [130]. Overexpressed PIAS1 is correlated with the development of colon cancer [143].

PIAS3	Upregulated	Increased expression of PIAS3 was observed in lung, breast, prostate, colon-rectum, and brain tumors [144].

PIAS4	Upregulated	Promotes the hypoxia-dependent EMT by regulating the transcriptional activity of SIRT1 [132]. Serves as an activator of hypoxia pathway in pancreatic cancer cells [133].

SENP1	Upregulated	Promotes the invasion of neuroblastoma by regulating the expression of MMP2, MMP9, and CDH1 [137]. Essential for the cell proliferation and migration of triple-negative breast cancer *in vitro* [145]. Increased level of SENP1 mRNA is correlated with cancer recurrence of bladder cancer [134].

SENP2	Downregulated	Limiting the expression of MMP13 and repressing the invasion and migration of bladder cancer [138]. Inhibiting the activation of NF-*κ*B pathway and improving the sensitivity of breast cancer cells to doxorubicin [146].

SENP3	Upregulated	Associated with the differentiation of oral squamous cell carcinoma [139]. Regulating cell proliferation by the deSUMOylation of PML under oxidative stress in colon adenocarcinoma [147]. SENP3 regulates the activity of nuclear Nrf2 under reactive oxygen species stress induced by cisplatin in laryngeal carcinoma [148].

SENP5	Upregulated	Subcellular location of SENP5 is associated with differentiation of oral squamous cell carcinoma [149]. SENP5 promotes breast cancer invasion by mediating TGF*β*RI SUMOylation [140].

**Table 3:** The mechanism of SUMOylation inhibitors.

Targets	Small molecular inhibitors	Mechanism
E1-SUMO thioester complex	Ginkgolic acid	The structures of these small molecular inhibitors are similar, and all include a carboxylic acid, which is essential for the direct and specific binding with SUMO E1, and can interrupt the formation of E1-SUMO thioester complex to block the SUMOylation.
	Anacardic acid	
	Kerriamycin B	

Ubc9-SUMO thioester complex	Spectomycin B1	Spectomycin B1 can directly bind with Ubc9 and interrupt the formation of Ubc9-SUMO thioester complex.

Ubc9-targeted protein	2-D08	2-D08 can block the SUMOylation without affecting ubiquitin. 2-D08 mainly interrupts the transfer of SUMO1 from Ubc9 to the targeted proteins.

## Abbreviations

SUMO: Small ubiquitin-like modifier
SUMO E1: SUMO activating enzyme
SAE1: SUMO activating enzyme subunit 1
SAE2: SUMO activating enzyme subunit 2
SUMO E2: SUMO conjugating enzyme
Ubc9: Ubiquitin conjugating enzyme
SUMO E3: SUMO ligase
SIM: SUMO interaction motif
PTM: Posttranslational modification
SENPs: *Sen*trin-specific proteases
Ubl proteins: Ubiquitin-like proteins
NF-*κ*B: Nuclear factor kappa light chain enhancer of activated B cells
I*κ*B*α*: Nuclear factor kappa light chain enhancer of activated B cells inhibitor *α*
PCNA: Proliferating cell nuclear antigen
PIAS: Protein inhibitor of activated STAT
TDG: Thymine-DNA glycosylase
NPC: Nuclear pore complex
RanGAP1: Ran GTPase-activating protein 1
pVHL: von Hippel-Lindau protein
HIF-1*α*: Hypoxia-inducible factor 1*α*
ATM: Ataxia telangiectasia mutated kinase
NEMO: NF-*κ*B essential modulator
EMT: Epithelial-mesenchymal transition
VEGF: Vascular endothelial growth factor
HCC: Hepatocellular carcinoma
ROS: Reactive oxygen species
TFAP2A: Activating enhancer binding protein 2 alpha
BRCA1: Breast cancer 1 protein
PTEN: Phosphatase and tensin homolog
PI3K: Phosphatidylinositol-3-kinase
PIP3: Phosphatidylinositol-3,4,5-triphosphate
pRB: Retinoblastoma tumor suppressor protein
MMP: Matrix metalloproteinase
MM: Multiple myeloma
SCLC: Small cell lung cancer
SAMe: S-Adenosyl methionine
MTA: 5′-Methylthioadenosine
PML: Promyelocytic leukemia protein
NB: Neuroblastoma
OSCC: Oral squamous cell carcinoma
TGF*β*RI: Type I transforming growth factor-*β*.
